# Cutaneous Adverse Events of Systemic Melanoma Treatments: A Retrospective Single-Center Analysis

**DOI:** 10.3390/ph16070935

**Published:** 2023-06-27

**Authors:** Lukas Kraehenbuehl, Stephanie Schneider, Laura Pawlik, Joanna Mangana, Phil Cheng, Reinhard Dummer, Barbara Meier-Schiesser

**Affiliations:** Department of Dermatology, University Hospital Zurich (USZ), University of Zurich (UZH), 8091 Zurich, Switzerlandjoanna.mangana@usz.ch (J.M.);

**Keywords:** melanoma, adverse event, toxicity, oncodermatology, targeted therapy, immunotherapy, immune-related

## Abstract

Recent progress in the treatment of advanced melanoma has led to the improved survival of affected patients. However, novel treatments also lead to considerable and distinct skin toxicity. To further characterize cutaneous adverse events (AE) of systemic treatments, we conducted a single-center retrospective study of biopsy-proven cutaneous adverse events of melanoma treatment over a period of 10 years at the University Hospital of Zurich, Switzerland. In 102 identified patients, 135 individual skin AEs developed. Immune checkpoint blockade (ICB) was causal for 81 skin AEs, and 54 were related to targeted therapies (TT). Recorded types of skin AEs included lichenoid, maculopapular, acneiform, urticarial, panniculitis, folliculitis, psoriasiform, granulomatous, eczematous, and others. The incidence of skin AEs was higher with TT (18.54%) than with ICB (9.64%, *p* = 0.0029). Most AEs were low-grade, although 19.21% of AEs were common terminology criteria for adverse events (CTCAE) Grades 3 or 4. A large spectrum of skin AEs was documented during treatment of advanced melanoma, and distinct phenotypes were observed, depending on treatment classes. AEs occurred earlier during treatment with TT than with ICB, and distinct types of skin AEs were associated with respective treatment classes. This study comprehensively describes skin AEs occurring during systemic treatment for melanoma at a single center.

## 1. Introduction

The incidence of malignant melanoma, the most lethal type of skin cancer, has rapidly increased in the past few decades [1,2]. With one in 50 individuals developing melanoma, it is the fifth most frequently diagnosed cancer in Western populations (3).

Until 2010, chemotherapy with dacarbazine was the standard of care for patients with metastatic melanoma; however, the objective response rate (ORR) was limited [2]. Within the last decade, treatment of advanced melanoma has improved substantially. The 5-year overall survival (OS) for Stage IV disease increased from 8.8% with dacarbazine chemotherapy to 52% with a combined immunotherapy regimen of ipilimumab and nivolumab [3,4]. 

Today, the backbone of advanced melanoma treatment consists of two approaches: immune checkpoint blockade (ICB) and targeted therapy (TT). 

Currently approved immunotherapy regimens for melanoma from the adjuvant setting to metastatic disease target immune checkpoints, such as cytotoxic T-lymphocyte antigen 4 (CTLA-4, e.g., ipilimumab), programmed cell death protein receptor 1 (PD-1, e.g., pembrolizumab and nivolumab), its ligand PD-L1 (e.g., atezolizumab), and lymphocyte-activation gene 3 (LAG3, e.g., relatlimab). Specifically, CTLA-4 is expressed on T-cells, where it competes with CD28 for the binding of CD80/CD86, thereby dampening the second signal and reducing early T-cell activation. On the other hand, PD-1 is expressed on activated T-cells; its ligand PD-L1 (as well as its second ligand PD-L2) is expressed both on myeloid cells and certain tumor cells, amongst others. The binding of PD-1 leads to inhibition of the T-cell and can ultimately lead to apoptosis of the previously activated T-cell. Lastly, LAG-3 also is an inhibitory receptor expressed on T-cells; its binding to MHC Class II decreases T-cell activity. It supports chronic T-cell exhaustion. A further role has been suggested in the suppressive function of regulatory T-cells. In healthy systems, these checkpoints prevent damage to healthy tissue from autoimmune disease. Whereas CTLA-4 regulates the initial phase of a T-cell response, PD-1 dampens the activity of effector T-cells. This dampening prevents melanoma from circumventing the endogenous immune system by inhibiting a repressor of immune activation [5]. Additional immunotherapy approaches are under investigation [6] or have recently been FDA-approved [7]. 

Targeted therapy, on the other hand, is based on aberrant signaling in the mitogen-activated protein kinase (MAPK)/extracellular signal-regulated kinases (ERK) pathway. Between 40 and 60% of melanomas harbor a targetable mutation in the gene BRAF, e.g., BRAF V600E [8,9]. Both BRAF and MEK are tyrosine kinases of central importance in the MAP kinase pathway, promoting tumor proliferation. These enzymes are amenable to inhibition through targeted small molecules. Inhibition of BRAF, as well as downstream MEK, suppresses this driver mechanism in melanoma proliferation. 

However, these novel treatment options, whilst improving patients’ survival, are frequently associated with distinct toxicities. In particular, cutaneous toxicity is amongst the most frequent adverse events in systemic melanoma treatment [10,11,12]. Whereas in pivotal prospective clinical trials ‘pruritus’ or ‘rash’ is reported with frequencies up to 40% [4], cutaneous adverse events are not systematically further described. 

Particularly well-described skin AEs of melanoma treatments include lichenoid immune-related cutaneous adverse events (ircAE), associated with PD-1/PD-L1 blockade [13], as well as acneiform rashes related to MEK inhibition [12]. More broadly, pruritus, maculopapular, eczematiform, and psoriasiform rashes are frequently described with ICB [14]. Bullous ircAE has a stronger association with PD-1- and PD-L1-based regimens but remains rather infrequent. When treating melanoma with ICB, vitiligo-like depigmentation (VLD) is a relatively frequent adverse event that is associated with improved response [15]. However, this skin AE is not only mostly restricted to melanoma patients but also thought to be the equivalent of an on-target effect. 

The side effect profiles of BRAF- and MEK-inhibitors differ in frequency depending on the regimen. The most commonly reported AEs with BRAF inhibitors are cutaneous toxicities (rash, photosensitivity, pruritus), fatigue, and arthralgia [16]. With MEK inhibitors, rash, diarrhea, and fatigue represent the most frequently described AEs [17]. The combination of BRAF- and MEK inhibitors does not lead to new toxicities but, intriguingly, reduces the incidence and severity of AEs observed with monotherapy regimens [18,19].

Whilst certain adverse events, such as lichenoid ircAE in PD-1-based ICB and acneiform rashes with TT, tend to be found consistently, the reported incidence of specific phenotypes varies widely between studies. Furthermore, most skin AEs of melanoma treatment reported in the literature are not biopsy-proven. Thus, to achieve a comprehensive and systematic characterization of all biopsy-proven skin AEs occurring due to melanoma treatments at a large comprehensive cancer center, we here report a single-center retrospective analysis of the last 10 years at the University Hospital of Zurich.

## 2. Results

In this retrospective analysis, we identified 135 individual skin AEs documented in 102 patients between January 2012 and December 2021. A sole skin AE occurred in 81 patients; 15 patients experienced two skin AEs; three patients with three skin AEs; and one patient each developed four, five, and six skin AEs, respectively. The average age at the timepoint of the development of the skin AE was 60.3 years (32.3–92.4 years). Seventy (69%) patients were male; 32 (31%) patients were female (see demographics Table 1).

Comprehensive treatment data were available for all patients receiving systemic treatment for melanoma since 2018. Between 2018 and 2021, 498 courses of ICB and 151 courses of targeted therapy were administered.

### 2.1. Types of Skin AEs

**Figure 1:**
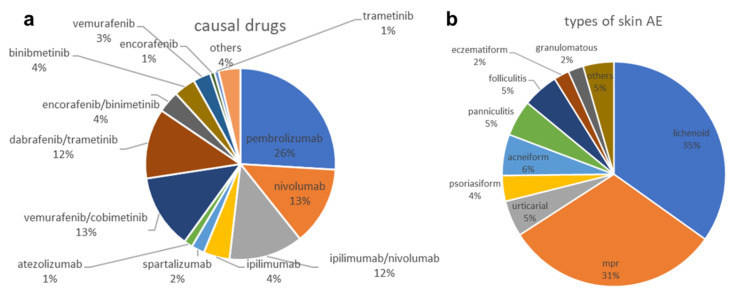
Overall distribution of drugs causing skin AEs (**a**) and subtypes of skin AEs recorded (**b**).

To characterize the clinicopathological type of skin AEs, we assessed overall incidences. Out of the 135 skin AEs, 47 (34.81%) were categorized as lichenoid, 42 (31.11%) as maculopapular, eight (5.93%) as acneiform, seven (5.19%) as urticarial, seven (5.19%) as panniculitis, seven (5.19%) as folliculitis, five (3.7%) as psoriasiform, three (2.22%) as granulomatous, three (2.22%) eczematous, and six (4.44%) others. The latter includes bullous (2), not classified (2), transient acantholytic dermatosis (e.g., Grover’s disease) (1), and pityriasis rubra pilaris (1) (Figure 1b). 

### 2.2. Drugs Causing Skin AEs

Out of the 135 skin AEs, 81 (60%) were attributed to immunotherapies, and 54 (40%) to targeted therapies (Figure 1a). ICB regimens (*n* = 81) included 35 (43.20%) pembrolizumab, 18 (22.22%) nivolumab, 17 (20.99%) combined ipilimumab and nivolumab, six (7.41%) ipilumumab, three (3.70%) spartalizumab, and two (2.47%) atezolizumab. Attributions to targeted therapies (*n* = 54) included 17 (31.48%) vemurafenib and cobimetinib, 16 (29.63%) dabrafenib and trametinib, five (9.26%) each of encorafenib and binimetinib, and binimetinib monotherapy, four (7.41%) of vemurafenib monotherapy, one (1.85%) case each of trametinib and of encorafenib, as well as five (9.25%) other tyrosine kinase inhibitors such as imatinib, naporafenib, and lenvatinib.

### 2.3. Incidence of Skin AEs by Treatment Class

Between 2018 and 2021, 498 courses of immunotherapy regimens were administered, as well as 151 courses of targeted therapies. Immunotherapies included 175 courses of combined CTLA-4 and PD-1 blockade, with 323 courses of anti-PD-1 monotherapy. TT included one course of BRAF inhibition alone and 150 courses of combined BRAF- and MEK inhibition. During this time, 76 biopsy-proven skin AEs were recorded, including 48 (*n* = 498, 9.64%) due to immunotherapies and 28 (*n* = 151, 18.54%) due to targeted therapies. Overall, patients treated with TT developed significantly more skin AEs (*p* = 0.0029).

### 2.4. Onset of Rash

**Figure 2:**
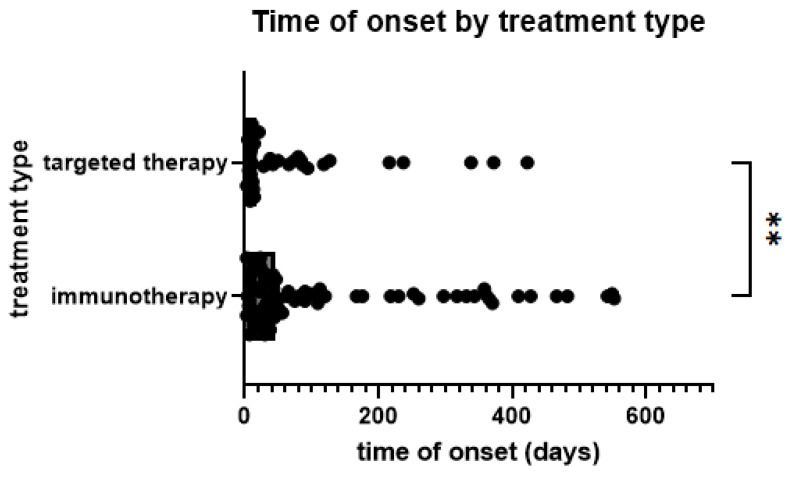
Time from start of treatment to onset of skin AE by treatment type; **: *p* < 0.005. (Mann–Whitney test, *p* = 0.0091).

**Figure 3:**
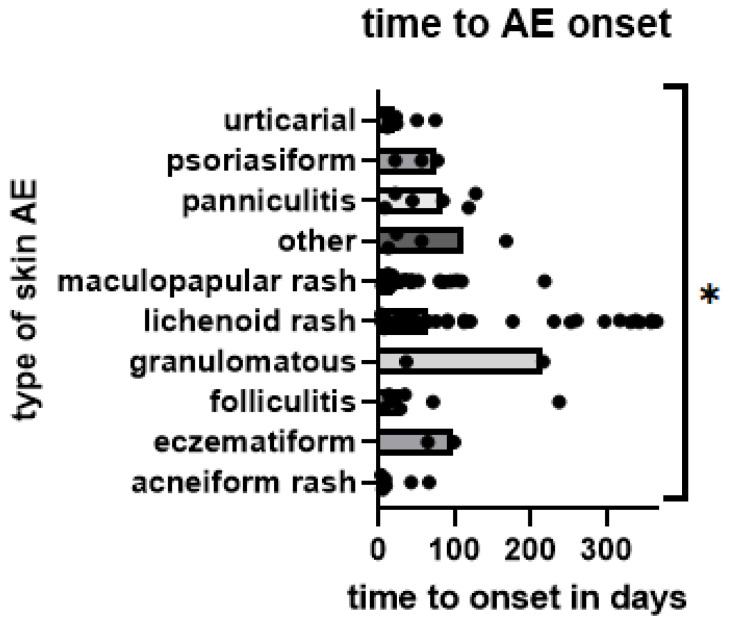
Time from start of treatment to onset of skin AE by type of skin AE; each AE is represented by one dot. Bars represent median time to onset per group; *: *p* < 0.05. (Kruskal–Wallis test, detecting significance in differences between medians of groups: *p* = 0.0144).

Skin AEs occurred earlier in patients treated with TT (median 17.5 days, mean 98.20 days) than with ICB (44.5 days and 208.70 days, *p* = 0.0091) (Figure 2). Comparing different types of skin AEs, time to onset varied significantly, with acneiform (median 8.5 days), maculopapular (18 days), and urticarial (22 days) occurring earliest. In contrast, granulomatous (median 216 days), eczematiform (99 days), panniculitis (84 days), and lichenoid (65 days) occurred later (Figure 3). However, pairwise comparisons of smaller groups should be avoided due to low respective sample size.

### 2.5. Association of Skin AE Types with Treatment Class

Acneiform rashes were strongly associated with TT (87.5% of all cases). When comparing incidences, it was higher (2.65%) with TT than with ICB (0.20%, *p* = 0.0116). Most (89.36%) lichenoid rashes were attributed to ICB; when comparing incidences, lichenoid skin AEs occur more frequently (4.42%) during ICB than during TT (1.99%, *p* = 0.1739). However, this trend is not statistically significant. Biopsy-proven maculopapular rash occurred more frequently with TT (61.90%) than with ICB (38.10%). This frequency translates to an incidence of 9.93% with TT and 2.83% with ICB (*p* = 0.0010). Comparing immunotherapy regimens’ causality for lichenoid AEs, the incidence with PD-1 monotherapy (5.06%) was higher compared to combinations with CTLA-4 blockade (3.03%). However, this observation did not reach statistical significance (*p* = 0.356). 

### 2.6. Severity of Skin AEs

Overall, 45 (33.82%) AEs were of CTCAE Grade 1, 64 (47.06%) of Grade 2, 23 (16.91%) of Grade 3, and three (2.21%) of Grade 4. The latter included two severe MPR and one lichenoid AE overlapping with Stevens–Johnson syndrome, the latter associated with nivolumab, the two others with TT. High-grade skin AEs (CTCAE Grades 3 and 4) were infrequent and more strongly associated with TT than with ICB (5.30% vs. 1.41%, *p* = 0.0103). All Grade 4 AEs led to treatment discontinuation and required treatment with systemic corticosteroids. Out of 23 Grade 3 AEs, four (17.39%) cases led to permanent discontinuation of the cancer treatment causing the skin AE. Additionally, combined ipilimumab and nivolumab was resumed as nivolumab monotherapy due to the G3 AE in one (4.35%) case. Furthermore, in six (26.09%) cases, treatment was interrupted or discontinued due to other adverse events, progression of disease, or other causes unrelated to the skin AE. In two (8.70%) G3 skin AEs, treatment was temporarily interrupted for improved management. Overall, 13 (56.52%) cases of G3 skin AE were maintained on active treatment, accompanied by appropriate management that included systemic corticosteroids or semi-occlusive high-potency topical corticosteroids. Of note, Grade 1 and 2 skin AEs did not lead to treatment interruptions and were successfully managed with topical corticosteroids or calcineurin inhibitors. Additionally, a subset of acneiform rashes was managed with oral tetracyclines in addition to topical corticosteroids. 

## 3. Materials and Methods

To identify all cases of biopsy-confirmed skin AEs of systemic melanoma treatments, we queried the histology database ‘Dermapro’ (Institut für Medizinische Software, Saarbrücken, German) of the University Hospital of Zurich, for pathology reports of skin AEs reported between 1 January 2012 and 31 December 2021.

The following key words have been used for the search within the ‘Dermapro’ histopathology database: “ipilimumab”, nivolumab”, “pembroli-zumab”, “vemurafenib”, “cobimetinib”, “dabrafenib”, “trametinib”, “encorafenib”, “binimetinib”, “check*”, “Anti-PD-1”, “MEK*”, and “BRAF-Inhibitor” for the medications and “lichen*” or “maculopapular,” along with “drug reaction” and “rash”, along with “immunotherapy” for the type of skin AE. 

In our study, we included skin AEs from patients with diagnosed MM, treated with immunotherapy or targeted therapies as mentioned above. In addition, patients receiving new therapies such as atezolizumab or spartalizumab developing skin AEs were also included in our analysis. 

Ensuring comprehensive documentation, only patients treated at the University Hospital of Zurich were included, and a high likelihood of a causal relationship between melanoma treatment and rash, as assessed by both the treating dermatologist and the investigators (board-certified in dermatology, fellowship in oncodermatology), was additionally required. Causality and the type of skin AE were determined mirroring clinical decisionmaking. The history and clinical presentation were integrated with the histopathological information to determine the type of skin AE as well as causal relationship by assessing the exposure to incriminated cancer drugs and by ruling out differential diagnoses. 

Cutaneous AEs associated with tebentafusp—a novel bispecific fusion protein—were not included, as tebentafusp is directly targeting melanocytes; therefore, skin inflammation is an expected on-target effect and wanted in this treatment approach. Hence, tebentafusp-associated cutaneous events were excluded. In addition, vitiligo-like depigmentation and alopecia were not considered for this investigation. Vitiligo-like depigmentations have previously been described [15], and alopecia is not conventionally considered a skin AE. In addition, vitiligo does not require a biopsy for diagnostic purposes. As an additional precautionary measure, events occurring during clinical trials were only included if the trial was un-blinded at the time of chart reviews. 

The CTCAE scale was used to grade the severity of skin AEs. Grade 0 would be no adverse event, and Grade 5 would be death due to the adverse event, with mild (Grade 1) impairing of activities of daily life (ADL), medical intervention required (Grade 2), hospitalization required (Grade 3), and life threatening (Grade 4) in between. For cutaneous AE, when no additional information regarding complicating factors, e.g., impairment of ADL, is available, Grade 1 is less than 10% body surface area (BSA) affected; Grade 2 is 10–30% BSA; and Grade 3 will be more than 30% BSA [11].

For approximate incidence calculations, internal registry data for systemic treatment of melanoma (part of the multi-center Eumelareg registry) collected between January 2018 and December 2021 were used. 

The data were collected, and descriptive statistics were computed using Microsoft Excel (Microsoft Corporation, Seattle, WA, USA). Additional statistical analyses were conducted using Prism (version 9.4.1, Graphpad Software Inc., San Diego, CA, USA); *p*-values < 0.05 were considered statistically significant.

## 4. Discussion

In this single-center review of skin AEs during 10 years of systemic treatment for advanced melanoma, we identified 135 individual, biopsy-proven events of skin AE in 102 patients. The most frequent types of skin AE were lichenoid and maculopapular rashes. Overall, targeted therapies were associated with a higher incidence of skin AEs, including high-grade skin AEs. Furthermore, rashes occurred earlier in patients treated with TT. Acneiform but also maculopapular rashes were mostly associated with targeted therapies, whereas lichenoid rashes were mostly attributed to ICB.

Overall incidences of rashes due to immunotherapy of 13.4–15% [20,21] for anti-PD-1 monotherapy and 40.3% for combined PD-1 and CTLA-4-blockade [22] have been reported in pivotal trials. For combined targeted therapy with BRAF- and MEK inhibitors, incidences of any grade rash have been reported to be as high as 15 to 39% [23,24,25]. Therewith, incidences for both immune-related skin AEs and those occurring due to TT are considerably higher in clinical trials than in our retrospective dataset. This difference is consistent with the higher rates of adverse events documented because of the more stringent AE assessments in the clinical trials setting. Furthermore, requiring confirmatory biopsies additionally reduces the number of AEs included and further explains the trend towards higher CTCAE grades in our dataset when compared to the keystone clinical trials. This finding is further supported by an incidence of 5.3% high-grade skin AEs occurring with TT regimens. Additionally, our data is consistent in identifying a higher rate of skin AEs occurring in TT than in ICB. Regarding the latter observation, in contrast to immunotherapy, toxicity of targeted therapies is dose-dependent. It is therefore possible that the equilibrium between maximum anti-tumor efficacy and minimum toxicity has not been reached yet.

Most recorded skin AEs were mild to moderate (CTCAE Grades 1–2). Focusing on biopsy-proven AEs might have further contributed to a higher overall grade of AE than expected. However, with 26 total cases, severe (Grade 3 and 4) AEs are infrequent and include only three life-threatening Grade 4 AEs.

Our findings are in line with previous reports describing a strong association of acneiform rashes with targeted therapies [19] as well as an association of lichenoid AEs with ICB [13]. Furthermore, previously described phenotypes of immune-related cutaneous adverse events have been identified in our patient collective [14,26]. The comparative incidence of lichenoid immune-related cutaneous adverse events (ircAE) to other ircAEs is higher in our study than the average of published data [26]. This finding might be due to the challenging clinical distinction of lichenoid ircAE more often leading to diagnostic biopsies as well as clinical interest in this AE subtype. This observation is otherwise consistent with an important overall heterogeneity in currently published datasets.

In cohorts of ircAEs, pruritus on apparently unchanged skin is often the most frequently reported adverse event [14,26,27,28]. These cases are not captured in our analysis, as pruritus alone is not formally a rash and macroscopically unchanged skin would very rarely be biopsied. Furthermore, vitiligo-like depigmentation is described as an on-target effect in patients with melanoma in general and frequently with ICB [15]. However, we did not include this well-characterized adverse event in our cohort.

Squamoproliferative conditions, in particular keratoacanthomas, are frequently reported with BRAF inhibitors [29]. Such cases were not included here, as we were focusing on inflammatory adverse events (e.g., “rashes”). Lastly, alopecia [25] and palmoplantar keratoderma [30] were not reported in our dataset, which is likely due to both our inclusion criteria and general patient preference against scalp and palmoplantar biopsies.

A limitation of this study is its retrospective design. Whilst requiring histopathological confirmation strengthens the presented data, it introduces bias of selection for performing skin biopsies. This bias includes higher-grade rashes, persistent AEs, lesser-known AEs, and AEs of specific interest, whilst potentially underreporting shorter-lasting AEs such as urticarial and MPR. The value of this investigation is therefore in the robust characterization of biopsy-confirmed skin AEs, rather than in providing accurate incidences. The availability of comprehensive treatment data for comparison purposes for four years instead of the full 10 years might further limit the interpretation of incidences. However, 77 of 135 skin AEs occurred during this limited time window too, as the number of administered treatment courses increased yearly. 

Early and adequate management of cutaneous toxicity is crucial in order to maintain dose intensity of effective melanoma treatments whilst ensuring patients’ quality of life. Where available, guidelines or peer-reviewed recommendations, such as the EADV Task Force recommendations on managing immune-related cutaneous adverse events [27], should be followed.

All patients undergoing systemic treatment for melanoma should be counseled on the use of fragrance-free moisturizers and the avoidance of harmful sun-exposure, as well as other skin irritations. Management of skin AE should be individualized based on the clinicopathological type in each patient.

For most Grade 1 skin AEs, medium- to high-potency topical corticosteroids (limit to medium for the face) should be prescribed, with no interruption of cancer treatment. With Grade 2 AEs, topical corticosteroids (see above) are required, and the addition of a short course of oral corticosteroids (0.5–1 mg prednisolone equivalent/kg) should be considered. In addition, specific approaches based on the type of skin AE are recommended. For example, phototherapy with UVBnb or specific biologics (e.g., TNF-blockade or apremilast) will be preferred for psoriasiform skin AE. In the case of Grade 2 or higher pruritus, GABA analogues, such as pregabaline or gabapentine can be introduced starting at low initial doses [31]. In acneiform rashes, topical antibiotics such as clindamycin or oral tetracyclines will be prioritized over topical corticosteroids [32]. From CTCAE Grade 2, cancer treatment interruptions should be considered for refractory AEs. Most Grade 3 AEs will require treatment interruptions, and discontinuation should be weighed against careful re-challenge after resolution of the skin AE and within the individual patient’s context of other toxicities and available alternatives.

A thorough assessment and detailed characterization of skin AEs in each patient will further enable the use of recent, targeted management approaches. As such, dupilumab, an IL4 and IL13 antibody, has recently been described as a potentially effective and safe treatment for cutaneous immune-related adverse events, including eczematous, maculopapular, and other phenotypes. Out of 39 treated patients, 34 showed at least a partial improvement of their skin AE. In addition, a significant decrease in blood eosinophils and a statistically insignificant reduction in serum IgE was noted [33]. Other promising targeted approaches are under investigation.

To identify precise incidences of subtypes of skin AEs due to treatment for advanced melanoma, pivotal trials should include a regular skin assessment by a dermatologist with precise characterization of the AE. As an alternative, a systematic prospective “real live” investigation could systematically characterize and document skin AEs prospectively in cohorts of patients treated for melanoma.

Early detection and appropriate phenotypic and histological classification are essential for timely and efficacious management of skin AEs of melanoma and of cancer treatments in general [34,35,36,37]. This treatment can improve a patient’s quality of life and the maintenance of the dose-density of cancer treatments, ultimately improving survival. Regular inspections of the entire skin in any patient treated for advanced melanoma is mandated. In addition to screening for secondary primary melanomas [38] and cutaneous metastases, this investigation demonstrates that skin AEs can appear as late as months to years after treatment starts.

## 5. Conclusions

This single-center retrospective analysis identified skin AEs occurring during treatment for advanced melanoma. Targeted therapies showed a stronger association with skin AEs, in particular with acneiform but also maculopapular rashes as well as severe AEs. ICB led to skin AEs less frequently; lichenoid rashes were the most frequently identified subtype. Skin AEs are an important complication to this potentially life-saving treatment and need to be detected early and managed efficiently to maintain patients’ quality of life. This retrospective study investigates the characteristics and incidences of skin AEs occurring in a setting different from a prospective clinical trial. The findings should enable clinicians to better anticipate, and therefore detect and treat, skin AEs of melanoma treatments. Further prospective investigations should be undertaken to ascertain the exact incidence of skin AE types.

## Figures and Tables

**Table 1:** Demographics.

	Targeted Therapy	Immunotherapy
**sex**		
**f**	19	20
**m**	35	61
**age**	58	62
	min–max (33–90)	min–max (32–92)
**treatment line**		
**1**	13	41
**2**	20	28
**3**	13	9
**4+**	8	3

## Data Availability

Not applicable.
